# Molecular identification of *Borrelia* and *Rickettsia* in hard ticks infesting domestic and wild animals in Cameroon

**DOI:** 10.1016/j.parepi.2025.e00475

**Published:** 2025-12-26

**Authors:** Archile Paguem, Pierre Kamtsap, Kingsley Tanyi Manchang, Alfons Renz, Sabine Schaper, Gerhard. Dobler, Robert E. Rollins, Lidia Chitimia-Dobler

**Affiliations:** a Department of Veterinary Medicine, Faculty of Agriculture and Veterinary Medicine, University of Buea, Cameroon; b Department of Comparative Zoology, Institute of Evolution and Ecology, University of Tübingen, 72076 Tübingen, Germany; c Bundeswehr Institute of Microbiology, Neuherbergstrasse 11, 80937 Munich, Germany; d Department of Parasitology, Institute of Zoology, University of Hohenheim, Stuttgart, Germany; e Institute of Avian Research “Vogelwarte Helgoland”, An der Vogelwarte 21, 26386 Wilhelmshaven, Germany; f Fraunhofer Institute of Immunology, Infection and Pandemic Research, Munich, Penzberg, Germany; g Experimental Parasitology, Department of Veterinary Sciences, Faculty of Veterinary Medicine, Ludwig-Maximilians-Universität, LMU, Munich, Germany

**Keywords:** Hard ticks, *Borrelia* spp., *Rickettsia* spp., Cameroon, Domestic and wild animals

## Abstract

Ticks are blood-sucking arthropods which can vector various, pathogenic microorganisms between humans and domestic or wild animal hosts. In Cameroon, little is still known about the diversity of ticks and tick-borne pathogens found feeding on these various hosts. This study investigates the frequency of positive pools of *Borrelia* spp. and *Rickettsia* spp. in 415 DNA pools arising from 1148 collected ticks belonging to five genera and twenty-five tick species collected from both domestic and wild animals in Cameroon. Tick species were identified morphologically and confirmed molecularly when necessary. All tick pools were tested for *Rickettsia* spp. and *Borrelia* spp. using molecular methods of which 18.01 % and 10.38 % of tick pools tested positive for *Rickettsia* or *Borrelia* DNA, respectively. This is the first *Borrelia* spp. detection in ticks collected from wild animals in Cameroon. Three species of *Rickettsia* were found in ticks feeding on domestic animals, namely, *Rickettsia africae, Rickettsia aeschlimannii*, and *Rickettsia massiliae. Borrelia* spp. in Cameroon are closely related to *Candidatus* Borrelia javanensis from China, as well as *Candidatus* Borrelia africana and *Candidatus* Borrelia ivorensis from the Ivory Coast. Although the risk this *Borrelia* species could pose to humans or animals is currently not known, both *Rickettsia* species are known to cause human disease warranting continuous monitoring and future research to determine the overall public health risk these microorganisms could pose.

## 1 Introduction

Ixodid ticks are obligate blood-feeding arthropods, which parasitize every class of terrestrial vertebrate in the world (Sonenshine and Roe, 2013). They primarily parasitize wild animals, with only about 10 % of species feeding on domestic animals (Sonenshine and Roe, 2013; Lane and Crosskey, 1993). There are currently 762 recognized species with 17 extant genera in the family Ixodidae which are divided into major two groups, the Prostriata and the Metastriata (Guglielmone et al., 2023; Barker et al., 2024; Kelava et al., 2024). Ticks are also known to harbour, and in some cases vector, a multitude of microorganisms such as viruses, bacteria, protozoa, and helminths (Jongejan and Uilenberg, 2004). Wild animals play a significant role as reservoirs for many pathogens, which can be easily spread to domestic animals or humans via infected tick bites (Liyanaarachchi et al., 2015; Ruiz-Fons and Gilbert, 2010). The resurgence of several tick-borne diseases continues to pose public health concerns and economic impacts worldwide (Abanda et al., 2019; Kuehn, 2019; Wesołowski et al., 2014; Balinandi et al., 2020; Adams, 2016). Although the significance of tick species as disease vectors infesting livestock, pet and humans is well-established, there are still much unknown with respect to tick diversity and host distribution on wildlife (Liyanaarachchi et al., 2015; Schulz et al., 2021) especially in many African countries.

Throughout Africa, different tick species are known to transmit tick-borne pathogens such as piroplasmoses caused by the protozoans belonging to the genera *Babesia* and *Theileria*, bacterial infections caused by species belonging to various genera (*Anaplasma*, *Borrelia*, *Ehrlichia*, *Rickettsia*), and also many viral diseases such as Crimean-Congo haemorrhagic fever (Abanda et al., 2019; Schulz et al., 2021). Members of the genus *Rickettsia*, such as the species *Rickettsia africae*, *Rickettsia aeschlimannii*, and *Rickettsia massiliae*, are known as the causative agent of African tick bite fever or spotted fever rickettsiosis. The most prevalent species in sub-Saharan Africa is *R. africae*, where certain tick species of the genus *Amblyomma* act as the main reservoirs and vectors (Kelly et al., 1996; Paguem et al., 2023; Parola et al., 2001; Vanegas et al., 2018). This infection has been reported with high seroprevalence in sub-Saharan African countries including Cameroon (11.9 %–51.8 %) (Ndip et al., 2004; Mediannikov et al., 2010). *Rickettsia aeschlimannii* was first identified in a patient returning from Morocco (Raoult et al., 2002). In this country, it was first isolated from *Hyalomma marginatum* ticks (Parola et al., 2005) but has also been reported by PCR in other *Hyalomma* species including *Hy. rufipes* and *Hy. truncatum* ticks collected from livestock in North Africa (Kernif et al., 2012). In West Africa, *R. aeschlimannii* was also detected in 15 % to 95 % of *Hy. rufipes* from Mali, Niger, Senegal, Nigeria, and Cameroon (Mediannikov et al., 2010, Mediannikov et al., 2012, Mediannikov et al., 2013; Parola et al., 2001; Parola, 2006; Vanegas et al., 2018; Diarra et al., 2017; Ngnindji-Youdje et al., 2022). Since its description in 2005, *R. massiliae* infections in humans have been confirmed in Europe and South America (Parola, 2006; Parola et al., 2008, Parola et al., 2013; Garcia-Garcia et al., 2010; Cascio et al., 2013). *Rickettsia massiliae* is thought to be associated with *Rhipicephalus* tick species and was detected by PCR in *Rhipicephalus* spp. from Côte d'Ivoire (Berrelha et al., 2009), *Rhipicephalus guilhoni* from Senegal (Mediannikov et al., 2010), *Rhipicephalus senegalensis* from Guinea (Mediannikov et al., 2012), *Rhipicephalus eversti eversti* from Nigeria (Reye et al., 2012) as well as *Rh. lunulatus* and *Rh. muhsamae* from Cameroon (Ngnindji-Youdje et al., 2022*)*.

In addition to rickettsioses, ixodid ticks can act as the vectors of various *Borrelia* species which, in some cases, can lead to diseases in humans. They are traditionally classified into the Lyme borreliosis (LB) group, the relapsing fever (RF) group and recently described third group of reptile/monotreme-associated borreliae (Margos et al., 2018). The causative agents of LB are ecologically associated with the ticks of the genus *Ixodes* and are predominantly found in the temperate northern hemisphere (Barbour, 1998). In contrast, RF group *Borrelia* are mostly associated with soft ticks and found in subtropical regions worldwide (Cutler et al., 2009; Trape et al., 2013). Relapsing fever is one of the most common diseases in several African regions including Senegal (Vial et al., 2006; Parola et al., 2011) and east African countries (Cutler et al., 2010). It is caused by different *Borrelia* species such as *Borrelia hispanica*, *Borrelia duttonii*, and *Borrelia crocidurae*. *Borrelia hispanica* was recently detected in 11.6 % to 20 % of *Ornithodoros* ticks from northern Africa (Trape et al., 2013; Sarih et al., 2009; Trape et al., 1996). *Borrelia crocidurae* is responsible for tick-borne relapsing fever in West Africa. In Ethiopia, a *Borrelia* spp. was identified by PCR in 7.3 % of *Amblyomma cohaerens* (Mediannikov et al., 2013). Phylogenetically, this *Borrelia* sp. was placed in an intermediate position between Lyme disease and relapsing fever groups and suggested to potentially belong to the reptile associated *Borrelia* (Mediannikov et al., 2013). The reptile-associated borreliae were described so far only from *Amblyomma*, *Hyalomma*, *Bothriocroton*, and *Ixodes* genera, mainly associated with reptiles and echidna (Takano et al., 2010; Takano et al., 2011; Jiang et al., 2021; Loh et al., 2017). The extent of these species' distribution though in other African countries, such as Cameroon, remains unclear.

In Cameroon, so far, 53 ixodid tick species are known from samples collected on domestic and wild animals (Ragenau, 1951; Ragenau, 1953; Morel and Mouchet, 1958; Morel and Mouchet, 1965). Most studies have focused on ticks parasitizing livestock (Bayemi, 1991; Awa et al., 2015; Silatsa et al., 2019; Vanegas et al., 2018; Ngnindji-Youdje et al., 2022) with fewer studies though reporting tick species from wildlife (Paguem et al., 2023; Morel and Mouchet, 1958). To date, however, the existence and/or prevalence of tick-borne associated pathogens (e.g., *Rickettsia* spp., *Borrelia* spp.) arising from ticks feeding on wild/domestic animals still remains poorly understood in this country. Our study aims to provide the first data screening for one such potential pathogen (*Borrelia*) in Wild animals and to provide complementary data on *Rickettsia* in ticks collected from both domestic and wild animals in Cameroon to provide a better understanding of the epidemiology of these pathogens in Cameroon.

## 2 Material and methods

### 2.1 Study sites, tick sampling, and species identification

**Fig. 1:**
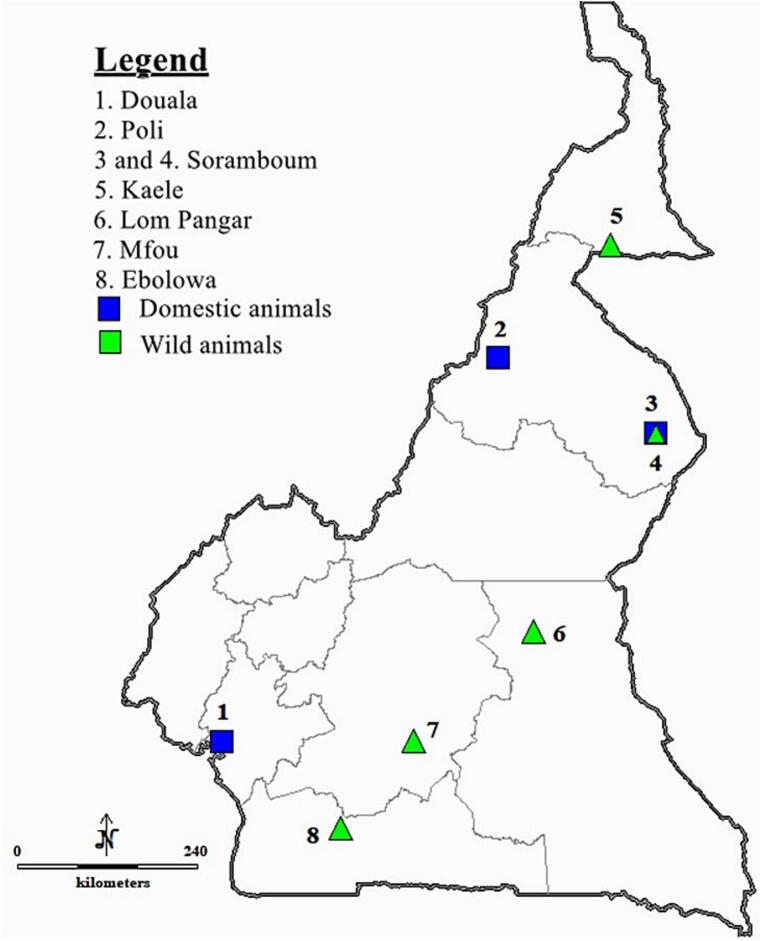
Map of the study area. The sampling areas (blue square and green triangle) were located in different areas of Cameroon. (For interpretation of the references to colour in this figure legend, the reader is referred to the web version of this article.)

Between June 2020 and November 2021, ticks were collected from domestic animals (cattle, goats, sheep, donkeys, dogs, and pigs) in three areas (Poli, Soramboum, and Douala) and from wild animals (*Phataginus tricuspis* (white-bellied pangolin), *Varanus niloticus* (Nile monitor), *Atelerix albiventris* (four-toed hedgehog), *Cephalophus rufilatus* (red flanked duikers), antelopes, *Lepus victoriae* (African savanna hare), *Cercopithecus* sp., *Caracal aurata* (African golden cat), *Mastomys natalensis*, *Civettictis civetta* (African civet), *Phacochoerus africanus* (warthog), *Python sebae* (African rock python), and *Atherurus africanus* (African brush-tailed porcupine)) sold in the bush meat markets in five localities (Kaele, Soramboum, Lom Pangar, Mfou, and Ebolowa) (Fig. 1). The information on tick species infesting wild animals and the *Rickettsia* species detected in the respective ticks has been previously published (Paguem et al., 2023). These samples are considered herein as at that time they were not screened for *Borrelia* spp. Selected animals were thoroughly examined for attached ticks and all the visible ticks were collected using pointed forceps in a manner as not to damaged them, and kept in vials containing 70 % ethanol until identification. All ticks were identified to the species level using morphological characteristics described by (Hoogstraal, 1956; Matthysse and Colbo, 1987; Apanaskevich et al., 2008; Voltzit and Keirans, 2003; Walker, 2014; Walker et al., 2000; Tomlinson and Apanaskevich, 2019; Arthur, 1965; Bakkes et al., 2020). Some tick species were confirmed by amplifying the 16S rRNA gene as described by Halos et al. (2004). Tick sequence data generated from wild animals was previously published in Paguem et al. (2023).

### 2.2 RNA/DNA extraction

Total RNA/DNA was extracted using the MagNA Pure LC RNA/DNA Kit (Roche, Mannheim, Germany) in a MagNA Pure LC instrument according to the manufacturer's instructions. DNA was extracted from individual ticks (if a single tick was found on a single host or a single life stage of a respective tick species) or pools (2–10 ticks per pool, if ticks belonged to the same species, developmental stage and were collected from the same animal). From the 1148 ticks collected this corresponded to 415 DNA pools which were screened for microorganisms in the PCR analyses described below. The extracted total DNA was stored at -80 °C until PCR analysis.

### 2.3 PCR amplification of *Rickettsia* spp and *Borrelia* spp

*Rickettsia* spp. DNA was analysed using a pan-*Rickettsia* real-time RT-PCR to amplify part of the *gltA* gene (Wölfel et al., 2008), ticks tested positive for rickettsiae were identified down to Rickettsia species level by PCR amplification of 23S—5S intergenic spacer region (Chitimia-Dobler et al., 2018).For this purpose, primers 23S and 23S rev and the thermoprofile of a previously published method (Jado et al., 2006) were modified to achieve optimum sensitivity. Briefly, 5 μl DNA, 0.5 μM Primer 23S for and 23S rev (Table 1), 1 U Platinum® Taq DNA Polymerase High Fidelity (Invitrogen), 1× reaction buffer, and a final concentration of 4 mM MgSO4 were added to a final volume of 50 μl per reaction. Initial denaturation at 95 °C for 2 min was followed by 45 cycles at 95 °C for 30 s, 30 s at 58 °C, and 30 s at 68 °C and a final extension at 68 °C for 10 min. The obtained DNA amplicons were visualized by gel electrophoresis.

**Table 1:** Primers and probes used for molecular investigation of tick species and their pathogens.

Genus	Primer	Target gene	Primer sequence (5′-3′)	Annealing T (°C)	Amplicon size (bp)	References
Ticks	TQ16S + F1	16S rRNA	CTGCTCAATGATTTTTTAAATTGCTGTGG		320	Halos et al. (2004)
	TQ-16S-2R		ACGCTGTTATCCCTAGAG			
*Rickettsia*	RH314:	gltA	AAACAGGTTGCTCATCATTC			Wölfel et al. (2008)
	RH654:		AGAGCATTTTTTATTATTGG		Not applicable	
*Rickettsia*	23S for	23S–5S intergenic spacer region	GATAGGTCGGGTGTGGAAGCAC			Chitimia-Dobler et al. (2018)
	23S rev		GGGATGGGATCGTGTGTTTCAC		378–532	
*Borrelia* outer	16S1A	16S rRNA	CTA ACG CTG GCA GTG CGT CTT AAG	63	1205	Richter and Matuschka (2006); Abanda et al. (2019)
	16S1B		AGC GTC AGT CTT GAC CCA GAA GTT			
*Borrelia* inner	16S2A	16S rRNA	AGT CAA ACG GGA TGT AGC AATAC	56	600–720	Richter and Matuschka (2006); Abanda et al. (2019)
	16S2B		GTT ATT CTT TCT GAT ATC AACAG			

For all PCR, standard procedures for PCR testing (three room concept, inclusion of positive and negative controls, extraction controls) were included in each run (Chitimia-Dobler et al., 2018).

Additionally, to detect *Borrelia* DNA, generic primers were used in a nested PCR targeting the 16S rRNA as described previously (Richter and Matuschka, 2006; Abanda et al., 2019). Briefly, the first reaction (25 μL final volume) contained 2 μM of each outer primer (Table 1), 0.2 mM dNTP mix, 0.5 U Go Taq DNA polymerase (Promega, Germany), 1× Go Taq buffer, and 1 μL of extracted DNA. Nuclease-free water was used as negative control and plasmid containing 10 copies of 16 s rDNA of *Borrelia burgdorferi* s.l kindly shared by colleagues from Freie Universität Berlin, Germany (Abanda et al., 2019) was used as positive control. The corresponding gene loci, primer pairs and annealing temperatures are shown in Table 1. PCR amplification was carried out as follows: initial denaturation step at 95 °C for 3 min, followed by 35 amplification cycles at 95 °C for 60 s, at 63 °C for 60 s, at 72 °C for 30 s, and final extension at 72 °C for 10 min (Master Cycler EP S Thermal Cycler®, Eppendorf, Hamburg, Germany). Thereafter, the second PCR reaction was carried out with 1 μL of first PCR product as template under the same cycling conditions as described above, except for an annealing temperature of 56 °C, and using the inner primer pairs (Table 1). All samples were visualized through electrophoresis on a 1.5 % agarose gel stained with Midori Green (Nippon Genetics Europe, Düren, Germany).

### 2.4 sequencing

The Rickettsia 23S—5S amplicon obtained were sequenced by Sanger sequencing (GATC Biotech, Konstanz, Germany) and Selected positive reactions were prepared following manufacturer's recommendations (Macrogen, Amsterdam, Netherlands) and sent for sequencing.

### 2.5 BLAST and phylogenetic analysis

All the sequences obtained were screened with BLASTn analysis (Altschul et al., 1990) and representative related sequences downloaded from GenBank (https://www.ncbi.nlm.nih.gov/ nucleotide).

Sequence data for *Rickettsia* 23S—5S intergenic spacer obtained from ticks collected in this study (*n* = 9) were compiled with reference data from GenBank and aligned using MUSCLE v3.8.425 (Edgar, 2004a, Edgar, 2004b) as implemented in Aliview v1.28 (Larsson, 2014). Phylogenetic reconstruction was performed in MrBayes v. 3.2.6 (Huelsenbeck and Ronquist, 2001; Ronquist et al., 2012) with ploidy set to haploid and a GTR (Tavaré, 1986) substitution model with inverse gamma-distributed rate variation. Three independent runs were launched and ran for two million generations. Convergence was checked with Tracer v. 1.7.1 (Rambaut et al., 2018). Consensus trees were built using the *sumt* command from MrBayes using a respective burn-in of 25 %. Convergence to a single topology in all three independent runs was checked manually in FigTree v. 1.4.4 (http://tree.bio.ed.ac.uk/software/figtree/). The *Rickettsi*a tree was rooted on the branch leading to the clade containing both *Rickettsia helvetica* reference sequences.

*Borrelia* species identity was determined through phylogenetic reconstruction based on the partial 16S rRNA sequences produced during this study. Sequences were compiled with GenBank references representing members of the known *Borrelia* clades (Lyme borreliosis, relapsing fever, reptile/monotreme-associated) and recently described *Borrelia* found in ticks from Africa. Sequences were then aligned using MUSCLE v3.8.425 (Edgar, 2004a, Edgar, 2004b) as implemented in Aliview v1.28 (Larsson, 2014). Phylogenetic reconstruction was performed in MrBayes v. 3.2.6 (Huelsenbeck and Ronquist, 2001; Ronquist et al., 2012) with ploidy set to haploid and a GTR (Tavaré, 1986) substitution model with inverse gamma-distributed rate variation. Three independent runs were launched and ran for five million generations. Convergence was checked with Tracer v. 1.7.1 (Rambaut et al., 2018). Consensus trees were built using the *sumt* command from MrBayes using a respective burn-in of 25 %. Convergence to a single topology in all three independent runs was checked manually in FigTree v. 1.4.4 (http://tree.bio.ed.ac.uk/software/figtree/). The 16S rRNA sequence from *Sediminispirochaeta smaragdinae* was included as an outgroup to root the tree.

Nucleotide sequence data reported in this paper are available in the National Center for Biotechnology Information (NCBI) GenBank™ databases under the accession numbers: PQ844815- PQ844825 for *Borrelia* and PV110811-PV110819 for *Rickettsia* sequences.

## 3 Results

### 3.1 Tick species and host range

In total, 1148 ticks (557 males, 471 females, 106 nymphs and 14 larvae) were collected from 117 domestics animals including cattle (*n* = 49), goats (*n* = 35), sheep (*n* = 7), donkeys (*n* = 1), dogs (*n* = 11), and pigs (*n* = 14) and from 166 out of 2000 wild animals examined. Infested wild animals belonged to thirteen species: *Phataginus tricuspis* (white-bellied pangolin, *n* = 48), *Varanus niloticus* (Nile monitor, *n* = 31), *Atelerix albiventris* (four-toed hedgehog, *n* = 25), *Cephalophus rufilatus* (red flanked duikers, *n* = 15), antelopes (n = 15), *Lepus victoriae* (African savanna hare, *n* = 9), *Cercopithecus* sp. (monkey, n = 7), *Caracal aurata* (African golden cat, *n* = 5), *Mastomys natalensis* (rodent, n = 4), *Civettictis civetta* (African civet, n = 3), *Phacochoerus africanus* (warthog, n = 2), *Python sebae* (African rock python, n = 1), and *Atherurus africanus* (African brush-tailed porcupine, n = 1) (all information about ticks collected from the 166 wild animals have already been published, see Paguem et al., 2023).

Based on morphological characteristics and 16S rDNA sequencing, 25 different tick species belonging to *Amblyomma, Haemaphysalis, Hyalomma, Ixodes,* and *Rhipicephalus* genera were identified. Overall, *Amblyomma compressum* (286/1148, 24.91 %) was the most common tick species collected and, although it was only found on wild animals, it represented almost half of the ticks exclusively collected from wild animals (286/686, 41.70 %, highly common on pangolins). The other two major tick species collected where *Rhipicephalus microplus* (197/1148, 17.16 %) and *Amblyomma variegatum* (192/1148, 16.72 %). The host ranges of the 27 species of ticks belonging to the family Ixodidae collected from domestic animals are shown in the Table 2 and information on host ranges for wild animals can be found in Paguem et al. (2023). Of the 25 tick species infesting 24 species of domestic and wild animal, then species, namely *Rh. guilhoni*, *Rhipicephalus annulatus*, *Rh. microplus*, *Rh. decoloratus*, *Rh. linnaei*, *Hy. truncatum*, *Hy. rufipes*, *Hy. nitidum*, *Rh. afranicus* and *A. variegatum* were common on domestic and wild animals. Eight tick species were only found in wild animals.

**Table 2:** Prevalence of *Rickettsia* spp. in tick collected from domestic animals. *Rickettsia* spp. detected in the ticks collected from wild animals are reported in Paguem et al. (2023).

Tick species	Tick life stages			Host	*Rickettsia* spp.	
	Males	Females	Nymphs		Pan*-Rickessia* positive/tick pool	*23S–5S* intergenic spacer region
*Amblyomma variegatum*	52	14	3	Cattle, goat,unidentified wild animals	14/20	*R. africae* (n = 3)
*Haemaphysalis leachi*		1		Dog	0/1	
*Hyalomma nitidum*		1		Dog	0/1	
*Hyalomma rufipes*	7	4		Cattle, sheep	1/6	*R. aeschlimannii* (n = 1)
*Hyalomma truncatum*	32	20		Pig, dog, cattle, unidentified wild animals, goats	5/13	*R. aeschlimannii* (n = 3)
*Rhipicephalus afranicus*	4	5		Goats	0/6	
*Rhipicephalus annulatus*		64		Sheep, goat, cattle, dog, unidentified wild animals	0/17	
*Rhipicephalus cuspidatus*		1		unidentified wild animals	0/1	
*Rhipicephalus decoloratus*		22		Goat, cattle, dog, unidentified wild animals	0/8	
*Rhipicephalus guilhoni*	8	3		Dog, goat, unidentified wild animals	0/7	
*Rhipicephalus lunulatus*	1	2		Goats, unidentified wild animals	0/3	
*Rhipicephalus microplus*	25	140		Sheep, goat, donkey, cattle, dog, unidentified wild animals	0/30	
*Rhipicephalus muhsamae*	33	12	1	Pig, dog, goat, cattle, unidentified wild animals	2/18	*R. massiliae* (*n* = 2)
*Rhipicephalus senegalensis*	3	3		Goats	0/3	
*Rhipicephalus* sp.			1	Cattle	0/1	
Total	165	292	5		22/135	

### 3.2 Frequency of positive pools of *Rickettsia* and *Borrelia* spp. in ticks collected from domestic and wild animals

**Fig. 2:**
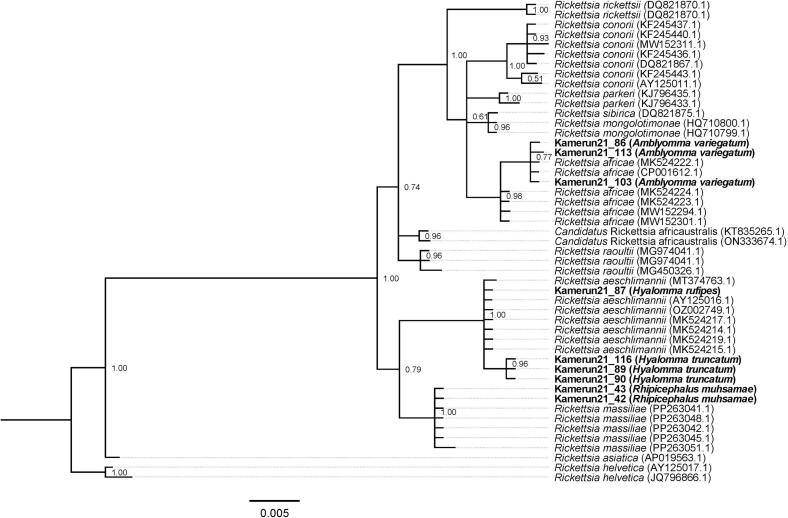
Phylogenetic reconstruction based on the 23S—5S intergenic spacer sequences for *Rickettsia* spp. amplified from ticks collected from wild/domestic animals in Cameroon. Samples sequenced in this study are shown in bold and GenBank accession numbers are included for all reference sequences. Phylogenetic reconstruction was performed in MrBayes v. 3.2.6 (Huelsenbeck and Ronquist, 2001; Ronquist et al., 2012) with ploidy set to haploid and a GTR (Tavaré, 1986) substitution model with inverse gamma-distributed rate variation. Three independent runs were launched and ran for 5 million generations. Convergence was checked with Tracer v. 1.7.1 (Rambaut et al., 2018). The phylogeny was rooted on the branch leading to the monophyletic clade containing both reference sequences for *R. helvetica*.

Briefly, sequencing results were obtained for 66 out of the 114 *Rickettsia*-positive ticks pools collected from wild animals as reported in Paguem et al. (2023). Three different *Rickettsia* species were identified in these samples with the most common species being *Rickettsia africae* 61/66, 92.4 %), with very few samples identified as *Rickettsia aeschlimannii* (3/66, 4.5 %) and *Candidatus* Rickettsia africaustralis (2/66, 3.0 %). *Rickettsia* sequences were obtained for nine of the 22 *Rickettsia*-positive ticks collected from domestic animals in this study (Table 2). These were identified as *R. aeschlimannii* (4/9, 44.4 %) in *Hy. rufipes* (n = 3) and *Hy. truncatum* (n = 1), *Rickettsia africae* (3/9, 33.3 %) in *A. variegatum* (n = 3), and *R. massiliae* was detected in *Rh. muhsamae* males (Table 2; Fig. 2). *Rickettsia africae* sequences were 99 % identical with the sequence MK524222 and MK524224 isolated from *A. lepidum* in Sudan*. Rickettsia aeschlimannii (*PV110813, PV110814 and PV110817) was 99.68 % identical with the sequences MT374764 and MK524216 from *Hy. rufipes* in Italy and Sudan respectively*. R. massiliae* (PV110818.1*)* was 100 % identical with PP263045, PP263050 and PP263048 from ticks in Greece*.*

**Fig. 3:**
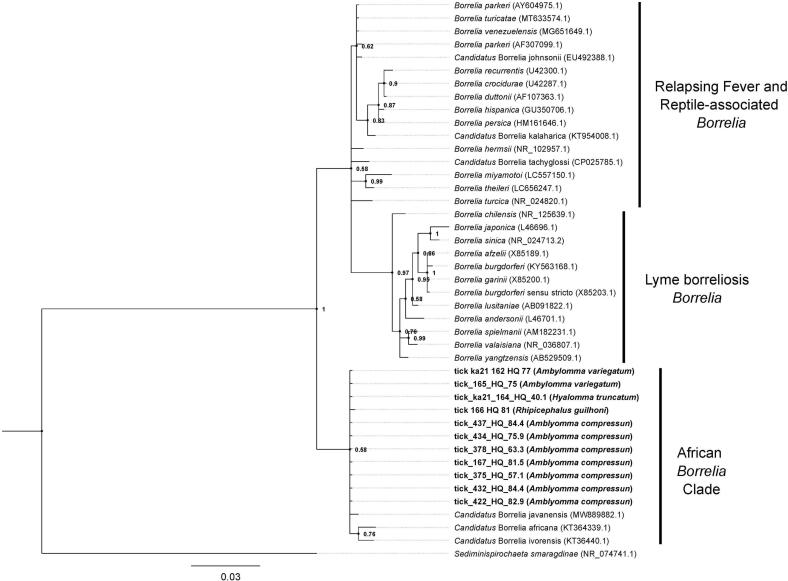
Phylogenetic reconstruction based on partial nucleotide sequences of the 16S rRNA (573 bp) genes of *Borrelia* spp. identified in ticks collected from domestic and wild animals in Cameroon. Samples sequenced in this study are shown in bold and GenBank accession numbers are included for all reference sequences. Phylogenetic reconstruction was performed in MrBayes v. 3.2.6 (Huelsenbeck and Ronquist, 2001; Ronquist et al., 2012) with ploidy set to haploid and a GTR (Tavaré, 1986) substitution model with inverse gamma-distributed rate variation. Three independent runs were launched and ran for 5 million generations. Convergence was checked with Tracer v. 1.7.1 (Rambaut et al., 2018). The 16S rRNA sequence from *Sediminispirochaeta smaragdinae* was included as an outgroup to root the tree. Numbers next to the nodes reports node probability and the scale bar is in substitutions per base pair.

Screening of all ticks for *Borrelia* spp. using nPCR, detected 49/415 (10.38 %) positive tick pools (Table 3). Ten out of 27 tick species tested positive to *Borrelia* spp.: namely *A. compressum* 30/111 (27.03 %), *A. flavomaculatum* 6/17 (35.29 %), *A. variegatum* 1/49 (2.04 %), *Hy. rufipes* 1/8 (12.5 %), *Hy. truncatum* 2/18 (11.11 %), *Rh. annulatus* 1/17 (5.88 %), *Rh. afranicus* 1/5 (20.00 %), *Rh. guilhoni* 2/2 (100 %), *Rh. lunulatus* 2/3 (66.67 %), *Rh. senegalensis* 3/3 (100 %). We succeeded in amplifying a fragment of the 16S rRNA in 11 ticks pools. Phylogenetic reconstruction based on the 16S rRNA fragment supported two major clades one containing all relapsing fever, reptile/monotreme-associated, and Lyme borreliosis species with a second clade containing newly described African *Borrelia* and all ticks sequenced in this study (Fig. 3). The split between these groups is highly supported with a node probability of 1 (Fig. 3). All sequenced *Borrelia* reported in this study show highest similarity to *Candidatus* Borrelia javanensis (GenBank: MW889882.1) isolated from pangolins in China.

**Table 3:** Prevalence of *Borrelia* spp. detected in ticks.

Tick species	Total tick pools	*Borrelia* spp. percentage of tick pools (%)	Host
*Amblyomma compressum*	111	30/111 (27.03 %)	White-bellied pangolins, Warthogs, Nile monitors, African brush-tailed, Porcupines, Monkeys, African golden cat, Antelopes, African civets
*Amblyomma flavomaculatum*	18	6/18 (33.33 %)	Nile monitors, Four toed hedgehogs, White bellied pangolins, Monkeys, Warthogs
*Amblyomma variegatum*	47	1/47 (2.13 %)	Nile monitor, Four toed hedgehog, Pigs, Dogs, Cattle, Goats, Un-identified wild animals
*Haemaphysalis leachi*	9		White-bellied pangolins, African civets
*Haemaphysalis camicasi*	1		Red flanked duiker
*Haemaphysalis houyi*	65		Nile monitors, Red flanked duikers, Monkeys, African savanna hares, Four toed hedgehogs, Antelopes, African rock python
*Haemaphysalis parmata*	2		Nile monitors, Antelope
*Hyalomma nitidum*	2		Red flanked duiker, Dogs
*Hyalomma rufipes*	8	1/8 (12.5 %)	Four-toed hedgehogs, Sheep, Cattle,
*Hyalomma truncatum*	18	2/18 (11.11 %)	Four-toed hedgehogs, Nile monitors, Pigs, Dogs, Cattle, Un-identified wild animals
*Ixodes moreli*	1		Antelope
*Ixodes rasus*	10		White-bellied pangolins, Four-toed hedgehogs, Warthogs, Monkeys
*Rhipicephalus microplus*	41		Four-toed hedgehogs, Nile monitors,
*Rhipicephalus decoloratus*	8		Cattle, Goat, Un-identified wild animals
*Rhipicephalus annulatus*	17	1/17 (5.88 %)	Cattle, Sheep, Goat, Un-identified wild animals
*Rhipicephalus afranicus*	19	1/19 (5.3 %)	Goat, Monkeys, African rock pythons, Red flanked duikers, Antelopes, Nile monitors, Four-toed hedgehogs
*Rhipicephalus guilhoni*	9	2/2 (100 %)	African savanna hares, Four-toed hedgehogs, Nile monitors
*Rhipicephalus lunulatus*	3	2/3 (66.67 %)	Goat and Un identified wild animals
*Rhipicephalus moucheti*	11		Red flanked duikers, Antelopes, Monkeys, Four-toed hedgehogs, African savanna hares, Nile monitors
*Rhipicephalus muhsamae*	10		Rats, Nile monitors
*Rhipicephalus linnaei*	2		Red flanked duikers, African savanna hares, Nile monitors
*Rhipicephalus senegalensis*	3	3/3 (100 %)	Goat
*Rhipicephalus* sp.	1		Cattle
Total	415	49/415 (11.81 %)

Two co-infections of *Rickettsia* and *Borrelia* (2/415; 0.42 %) were detected by nPCR. Co-infections involved the presence of an unidentified as *Borrelia* spp. closely related to *Candidatus* Borrelia javanensis with *R. aeschlimannii* in a single *Hy. rufipes* collected from a four-toed hedgehog and with *R. africae* in a single *A. flavomaculatum* collected from a Nile monitor.

## 4 Discussion

*Borrelia* and *Rickettsia* species in ticks collected from domestic and wild animals in Cameroon were investigated. The present study provides the first molecular proof for the presence of *Borrelia* spp. closely related to *Candidatus* Borrelia javanensis in ticks from wild animals in Cameroon.

Three *Amblyomma* species were recorded from domestic and wild animals, with *A. compressum* and *A. flavomaculatum* exclusively found on wild animals, whereas *A. variegatum* were found on both domestic and wild animals. Further description of tick diversity and host range in wild animals can be found in Paguem et al. (2023). The additional results from domestic animals show, that nine species, namely *Rh. guilhoni*, *Rh. annulatus*, *Rh. microplus*, *Rh. decoloratus*, *Rh. linnaei*, *Hy. truncatum*, *Hy. rufipes*, *Hy. nitidum*, and *A. variegatum* infested both domestic and wild animals. In this study, and as previously shown on cattle and small ruminants, three tick species were predominant: *Rh. decoloratus, Rh. microplus*, and *A. variegatum* (Awa et al., 2015; Silatsa et al., 2019*;* Ngnindji-Youdje et al., 2022)*.* This finding could suggest that wild animals that cohabit with domestic animals could additionally act as hosts of tick classically found on livestock (Pegram et al., 1987).

There are many reports about *A. variegatum* carrying *R. africae* from different African countries such as Sudan (Springer et al., 2020; Shuaib et al., 2020), Ethiopia (Tufa et al., 2021), and Cameroon (Ndip et al., 2004; Vanegas et al., 2018*;* Ngnindji-Youdje et al., 2022; Paguem et al., 2023). In the present study, *R. africae* was detected in *A. variegatum* collected from domestic animals and in *A. compressum* and *A. flavomaculatum* collected from wild animals (see Paguem et al., 2023). The high proportion of infested ticks from our data and previous reports could suggest that tick species from the genus *Amblyomma* play an important role as vector and reservoir of *R. africae* in the Afrotropical region*. Rickettsia aeschlimannii* was identified in two *Hy. rufipes* and one *Hy. truncatum* in the screened tick samples. Samples from this study however formed two distinct clades within the phylogeny. Even so, upon sequence comparison the sequences were over 99 % similar suggesting this structure could be due to local sequence polymorphisms instead of representing a novel *Rickettsia* lineage or species. *Rickettsia aeschlimannii* is a recognized human pathogen causing spotted fever and has been detected in different countries in sub-Saharan Africa in *A. variegatum, Rh. annulatus, Rh. evertsi evertsi, Rh. appendiculatus, H. rufipes*, and *H. truncatum* (Parola et al., 2013). *Rickettsia massiliae* was detected in two *Rh. muhsamae* males (one male tested individual and in one 10 male pool) collected from pigs in Sorrambum in the Mayo-Rey region. It was previously reported in ticks collected from cattle in the western region of this area. *Rickettsia massiliae* was detected in (16/27) *Rh. lunulatus*, *Rh. muhsamae* (1/10) (Ngnindji-Youdje et al., 2022) and in one *Rh. lunulatus* from Cameroon (Vanegas et al., 2018)*. Rickettsia massiliae* was reported from Côte d'Ivoire in *Rh. senegalensis* (33 %) and in *Rh. guilhoni* (22 %) from Senegal (Sarih et al., 2009; Trape et al., 1996; Georges, 2005). Further investigations are necessary regarding the species identity and phylogeny of these unidentified *Rickettsia* species.

The association of *Borrelia* spirochetes with ticks in Africa in general and in Cameroon in particular is still poorly understood. Until now only *Borrelia theileri*, a member of the tick-borne relapsing fever group was reported in one *Rh. microplus* tick collected in western region of Cameroon (Ngnindji-Youdje et al., 2022) and detected in cattle blood in the country (Abanda et al., 2019). In this study, *Borrelia* sp. was detected in 10.38 % of tick pools in three *Amblyomma* species (*A. compressum*, *A. flavomaculatum*, *A. variegatum*), two *Hyalomma* species (*Hy. rufipes*, *Hy. truncatum*), and five *Rhipicephalus* species (*Rh. annulatus*, *Rh. afranicus*, *Rh. guilhoni*, *Rh. lunulatus*, and *Rh. senegalensis*) collected from domestic and wild animals across different geographical areas. The BLAST and phylogenetic analysis of the successfully sequenced samples (*n* = 11), showed that these *Borrelia* sp. shared similarity and clustered with *Candidatus B. javanensis* detected in 3 % (12/227) of *Amblyamma javanense* ticks collected from pangolins (*Manis javanica*) in China (Jiang et al., 2021). Interestingly, most of the *Borrelia* spp. closely related to *Candidatus B. javanensis* positive samples were from *A. compressum* ticks collected feeding on pangolins. This could suggest that a potential association between *Borrelia* spp. closely related to *Candidatus B. javanensis* and pangolins could exist, although this would need to be stringently tested in lab-based studies*.* The clade within the phylogeny also contained other recently described *Borrelia*, namely, *Candidatus B. africana* and *Candidatus B. ivorensis* from *A. variegatum* in Côte d'Ivoire (Ehounoud et al., 2016). We detected *Borrelia* spp. closely related to *Candidatus B. javanensis* in *A. compressum*, *A. flavomaculatum*, in one *A. variegatum,* two *Hy. truncatum* and one *Rh. guilhoni* from Cameroon*.* Our study represents, to the best of our knowledge, the first report of *Borrelia* spp. closely related to *Candidatus B. javanensis* in *A. compressum* and *A. flavomaculatum.* Blast analysis of the 16S rRNA gene did show variation between the reference sequence for *Candidatus B. javanensis* from screened ticks collected in China although all sequences reported in this study clustered together with this reference sequence in the phylogenetic reconstruction. Our phylogenetic reconstruction differs slightly from previous reports of *Candidatus B. javanensis* suggesting that this and other African *Borrelia* may be separate from the known *Borrelia* clades (Jiang et al., 2021) although analysis including additional genetic markers is needed to support this. Further molecular investigation is needed, however, to better resolve the phylogeny by including analysis of additional marker genes (e.g., *flaB*, *gyrB*) genes or utilizing whole genome sequencing. Our results suggest the possibility of more complex vector pathogen-reservoir host interactions which need to be investigated in more detail. Additionally, the pathogenicity and zoonotic potential of the *Borrelia* reported here is not known and will need to be addressed in future studies.

## 5 Conclusions

The present study was carried out to determine which tick species infest domestic and wild animals in Cameroon and also to determine which are positive for *Borrelia* and *Rickettsia*. As far as it is known, this is the first kind of tick survey both on domestic and wild animals and their associated *Borrelia* and *Rickettsia* pathogens performed in Cameroon. In total, twenty-five tick species belonging to *Amblyomma*, *Haemaphysalis*, *Hyalomma*, *Ixodes*, and *Rhipicephalus* genera were collected from six domestic and thirteen wild animal species. Three species of *Rickettsia* were detected in ticks collected from domestic animals namely *R. africae*, *R. aeschlimannii*, and *R. massiliae*. Additionally, *Borrelia* spp. are reported for the first time. *Borrelia* species detected in ticks from wild and domestic animals in Cameroon are closely related to *Candidatus B. javanensis* from China, and from a clade with *Candidatus B. africana* and *Candidatus B. ivorensis* from Ivory Coast. Although this study is limited to the analysis of partial 16S fragment sequences *Borrelia* spp., robust multilocus (e.g., flaB, glpQ, gyrB) or whole genomic sequencing is needed for confirmation. Further studies should be encouraged to investigate the risk of tick-borne diseases, especially in relation to potential bovine borrelioses, in order to reduce potential risks in the region.

## CRediT authorship contribution statement

**Archile Paguem:** Writing – review & editing, Writing – original draft, Visualization, Validation, Investigation, Formal analysis, Data curation, Conceptualization. **Pierre Kamtsap:** Writing – review & editing, Writing – original draft, Visualization, Validation, Formal analysis. **Kingsley Tanyi Manchang:** Writing – review & editing, Writing – original draft, Methodology, Formal analysis. **Alfons Renz:** Writing – review & editing, Writing – original draft, Validation, Supervision, Conceptualization. **Sabine Schaper:** Writing – review & editing, Writing – original draft, Methodology, Formal analysis. **Gerhard. Dobler:** Writing – review & editing, Writing – original draft, Resources, Methodology, Conceptualization. **Robert E. Rollins:** Writing – review & editing, Writing – original draft, Software, Methodology, Formal analysis, Data curation. **Lidia Chitimia-Dobler:** Writing – review & editing, Writing – original draft, Visualization, Validation, Supervision, Resources, Methodology, Formal analysis, Conceptualization.

## Ethical statement

The study was approved by the National Institute of Agricultural Research for Development (IRAD) in Cameroon, which is the country's government institution for animal health and husbandry improvement. The ethical clearance number is CEIUD/371/01/2016/M.

## Funding statement

This research did not receive any specific grant from funding agencies in the public, commercial, or not-for-profit sectors.

## Declaration of competing interest

None.

## Data Availability

Data will be made available on request.
